# Dental Abnormalities in Osteogenesis Imperfecta: A Systematic Review

**DOI:** 10.1007/s00223-024-01293-2

**Published:** 2024-09-18

**Authors:** Laura Ventura, Sara J. E. Verdonk, Lidiia Zhytnik, Angela Ridwan-Pramana, Marjolijn Gilijamse, Willem H. Schreuder, Kirsten A. van Gelderen-Ziesemer, Ton Schoenmaker, Dimitra Micha, Elisabeth M. W. Eekhoff

**Affiliations:** 1 Department of Human Genetics, Amsterdam UMC Location Vrije Universiteit Amsterdam, Amsterdam, The Netherlands; 2 Amsterdam Reproduction and Development, Amsterdam, The Netherlands; 3 Amsterdam Movement Sciences, Amsterdam, The Netherlands; 4 Amsterdam Bone Center, Amsterdam, The Netherlands; 5 Department of Internal Medicine Section Endocrinology, Amsterdam UMC Location Vrije Universiteit Amsterdam, De Boelelaan 1117, Amsterdam, The Netherlands; 6 Department of Oral and Maxillofacial Surgery, Amsterdam UMC Location Vrije Universiteit Amsterdam, Amsterdam, The Netherlands; 7 Department Maxillofacial Prosthodontics, Stichting Bijzondere Tandheelkunde, Amsterdam, The Netherlands; 8 Department of Oral Diseases and Maxillofacial Surgery, Academic Centre for Dentistry Amsterdam (ACTA), Amsterdam, The Netherlands; 9 Medical Library, Vrije Universiteit, Amsterdam, The Netherlands; 10 Department of Periodontology, Academic Centre for Dentistry Amsterdam (ACTA), Amsterdam, The Netherlands

**Keywords:** Osteogenesis imperfecta, Dentinogenesis imperfecta, Collagen type I, Dental abnormalities

## Abstract

Osteogenesis imperfecta (OI) is a rare genetic disorder characterized by fragile bones and skeletal deformities. Individuals with OI may have dental abnormalities such as dentinogenesis imperfecta (DI) type I, malocclusions, and unerupted or missing teeth. This review comprehensively examines these dental abnormalities to assess their prevalence among the OI population and explore potential differences across different clinical types of OI and pathogenic variants. In accordance with the PRISMA guidelines, a systematic literature search in PubMed, Embase, and Web of Science was conducted that included articles up to June 2024. Out of 672 articles screened, 34 were included. The included studies confirmed that dental abnormalities are prevalent in OI, with DI prevalence ranging from approximately 20 to 48%. Those with a more severe skeletal phenotype (OI type III/IV) exhibited more dental abnormalities than those with a milder skeletal phenotype (OI type I). Notably, OI type V individuals generally do not have DI, although a few isolated cases have been reported. The prevalence of occlusion types varied: Class I occlusion ranged from 14.8 to 50% and Class II malocclusion ranged from 0 to 37.5%, while Class III malocclusion from 4.1 to 84%. This differs from the general population, where Class III malocclusion is typically the least common. Open bites, cross-bites, and unerupted and missing teeth are also commonly reported, particularly in OI types III and IV. This review emphasizes the need for comprehensive dental examinations in OI due to the high prevalence of dental abnormalities. Additionally, the review draws attention to the lack of clear guidelines for diagnosing DI.

**Supplementary Information:**

The online version contains supplementary material available at 10.1007/s00223-024-01293-2.

## Introduction

Osteogenesis imperfecta (OI) is a rare genetic disorder characterized by bone fragility and severe skeletal deformities, leading to increased susceptibility to fractures [1]. It affects approximately 1 in 15,000–20,000 individuals and is typically inherited through germline transmission [2]. In 85% of cases, OI is attributed to numerous monogenic variants in the *COL1A1* (OMIM #120150) or *COL1A2* (OMIM #120160) genes, which encode collagen type I, while about 15% of cases involve pathogenic variants in genes that commonly affect the biosynthesis pathways of collagen type I [2]. Beyond its impact on skeletal health, OI is also frequently associated with dental abnormalities that significantly affect the well-being of those affected [3]. Notably, since collagen type I is a critical component of teeth, pathogenic genetic variants causing a defect in collagen type I directly impact dental health. Therefore, approximately half of the individuals with OI experience some degree of dental abnormalities [4]. Additionally, due to the increased risk of fractures, dental attrition and enamel fractures are a common concern among OI patients [5].

**Fig. 1:**
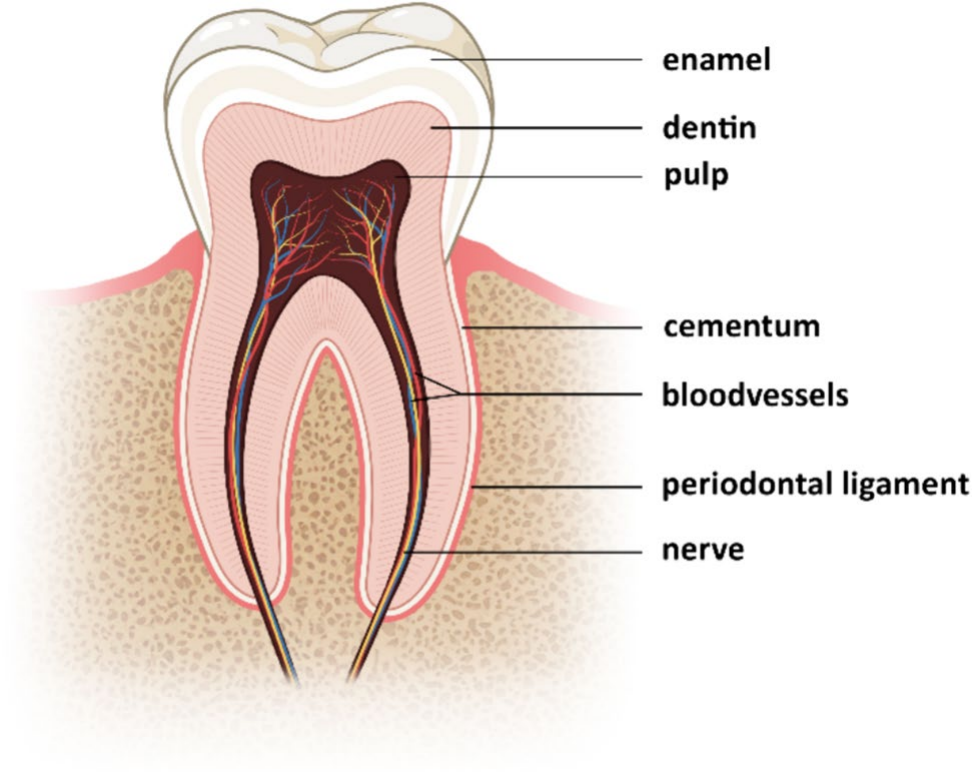
Cross-section of a molar showing the structures of the tooth. Created with Biorender.com and edited with Adobe Photoshop (Beta) version 25.11

Collagen type I, the most abundant type of collagen in the human body, is predominantly found in bones, skin, tendons, and ligaments, and in teeth. In teeth, collagen type I is primarily located in two main structures: the dentin and the periodontal ligament (Fig. 1) [6]. Dentin, located beneath the enamel and cementum, forms most of the tooth’s structure, supporting the enamel. Comprising 20% organic material, mostly collagen type I, the mechanical properties of dentin are vital for tooth strength [7]. The periodontal ligament, a specialized connective tissue encircling tooth roots, anchors them in the alveolar bone. It abundantly consists of collagen type I and connects the tooth cementum to the alveolar bone socket, providing crucial support against chewing forces. Although in smaller quantities compared to dentin and the periodontal ligament, collagen type I is also present in the cementum and pulp [8].

Dental abnormalities in individuals with OI can range from subtle variations in tooth morphology to severe defects in dentin formation [9]. Dentinogenesis imperfecta (DI) is a disorder characterized by disruptions in dentin development and is classified into three types (I, II, III), which are clinically, radiologically, and histologically similar but genetically distinct [8, 10]. Types II and III are not associated with a systemic disorder and, therefore, are not linked to OI. These types are inherited in an autosomal dominant manner and are associated with variants in the dentin sialophosphoprotein (DSPP) gene [11]. DI type I dental dysplasia, commonly seen in individuals with OI, is the only type of DI associated with this condition [12]. It is frequently associated with pathogenic variants altering the genes related to collagen type I production and transport [11]. Teeth with defective dentin may appear discolored and fragile. DI does not only affect the esthetics and function of the dentition but can also contribute to and coexist with oral health complications, such as increased susceptibility to dental caries, taurodontism (enlarged pulp chambers), malocclusions, and tooth agenesis [9, 13–16]. Radiographic features include bulbous crowns with significant cervical constrictions, and the pulp chambers become unrecognizable over time due to accumulation of secondary dentin [11, 17]. DI can affect both deciduous and permanent dentitions [5, 10, 18]. There are no established guidelines defining the criteria for DI diagnosis and not all individuals with DI exhibit identical characteristics [19]. In OI, occlusal abnormalities, or misalignment of dental arches, are common [20]. Tooth agenesis, the congenital absence of one or more teeth, is reported in individuals with OI, along with tooth loss due to extraction, trauma, and abnormal tooth eruption [10, 14, 16]. Monitoring and early intervention are crucial to minimize dental complications and improve oral health [19].

The skeletal phenotype of individuals with OI exhibiting collagen type I defects can vary widely, ranging from non-deforming OI (type I; OMIM #166200), to moderate OI (type IV; OMIM #166220), progressively deforming OI with short stature and severe bone deformities (type III; OMIM #259420) and perinatally lethal OI (type II; OMIM #166210), as categorized by the clinical Sillence classification [1]. OI Type V (OMIM #610967), which is distinctly marked by interosseous calcifications and hyperplastic callus formation, was added to this classification in 2000 [2, 21, 22]. These differences in phenotype are attributable to the specific pathogenic variants. Individuals with OI type I often carry a pathogenic variant caused by a quantitative defect that results from haploinsufficiency, while individuals with OI types II, III and IV typically harbor pathogenic variants that result in a structurally abnormal collagen type I protein, leading to a qualitative defect in collagen type I. OI type V is specifically caused by a pathogenic variant in the 5’ UTR region of the *IFITM5* (OMIM #614757) gene, which encodes a protein involved in bone formation and mineralization [2]. This wide phenotypic and genetic variability presents significant challenges in management and prognosis. This complexity is particularly evident in extraskeletal manifestations, such as dental abnormalities, which currently cannot be definitively linked to specific types of OI. Notably, dental issues seem to occur more often in individuals with OI types III and IV [5, 10].

This systematic review aims to expand upon the existing literature on dental issues related to OI subtypes by offering a comprehensive exploration. Besides DI, various tooth malformations and misalignment occur in OI patients, yet a detailed exploration of these manifestations is lacking. Therefore, the aim is to assess the prevalence of DI, malocclusions and missing teeth in individuals with OI and to investigate potential differences in dental abnormalities among clinical types of OI and associated pathogenic variants. In this review, only studies specifically focusing on dental abnormalities in individuals with OI are selected.

## Methods

### Literature Search

This review was conducted according to the Preferred Reporting Items for Systematic Reviews and Meta-Analyses (PRISMA) guidelines [23]. After several scoping searches, three bibliographic databases (Ovid/Medline, Embase.com, and Clarivate Analytics/Web of Science Core Collection) were searched for relevant literature from inception to June 6, 2024. Searches were devised in collaboration with a medical information specialist (KAZ). Search terms, including synonyms, closely related words, and keywords, were used as index terms or free-text words: “osteogenesis imperfecta” and “dental malformation”. The searches contained no date or language restrictions that would limit results to specific dates or language. Google Scholar, conference proceedings, and references of both included full-text studies and relevant systematic reviews were searched for additional relevant literature. The complete search strategy for each database can be found in the Supplementary material.

### Selection Process

Two reviewers (LV and SV) independently screened all potentially relevant titles and abstracts for eligibility. Differences in judgment were resolved through a consensus procedure. Studies were included when they met the following criteria: (i) studies containing patients with osteogenesis imperfecta; (ii) studies giving an adequate description of the dental abnormalities and/or the oral examination performed; (iii) studies published in English; (iv) full-text availability; and (v) case–control studies, cohort studies, and case series of at least 10 patients. The following exclusion criteria were used: (i) cases of OI type II (prenatally lethal form); (ii) studies with only previously described OI cases; (iii) studies focusing on animal models; and (iv) studies that exclusively involved individuals from a single family, to minimize the potential for biased data and to ensure a broader representation of the population under study.

### Quality Assessment

The full text of the selected articles was obtained for further review. Two reviewers (LV and SV) independently evaluated the methodological quality of the full-text papers using the Study Quality Assessment Tool created by NHLBI [24].

## Results

### Search Results

**Fig. 2:**
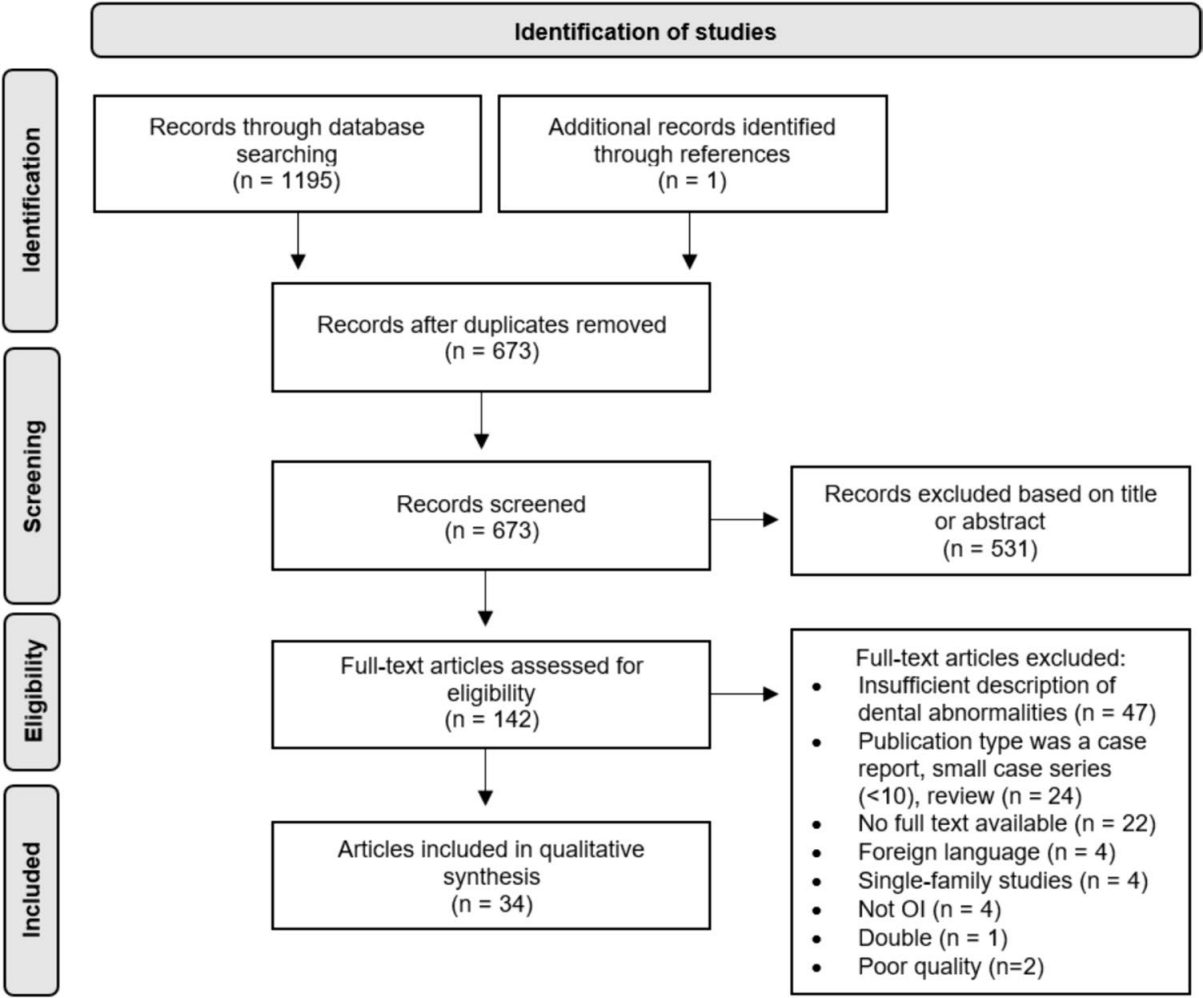
Flowchart of the search and selection procedure of studies

The literature search generated a total of 1195 references: 368 in Ovid/Medline, 497 in Embase.com, and 330 in Clarivate Analytics/Web of Science Core Collection. After removing duplicates of references that were selected from more than one database, 673 references remained. The flowchart of the search and selection process is presented in Fig. 2. Of the total 673 articles that were identified, 142 were included for full-text analysis. In total, 36 articles were included, all being large case series (*n* > 10), cohort studies, or case–control studies (Table 1) [3, 5, 10, 13–16, 18, 19, 25–49]. A quality assessment (Study Quality Assessment Tool by NHLBI [24]) of the included articles was performed by the two reviewers independently; 5.6% (*n* = 2) of the studies were classified as poor, 33.3% (*n* = 12) as fair; and 61.1% (*n* = 22) as good. Articles assessed as poor were excluded, resulting in 34 articles included in the qualitative synthesis.

**Table 1:** Studies on dental abnormalities in osteogenesis imperfecta which are included in this review

First author (year)	Country of included patients	Inclusion years	Study design	Individuals assessed for dental manifestations, *n*	Females, *n* (%)	Age range, *years*	OI types included in the study (Sillence classification)	Dental abnormalities studied
Andersson (2017) [25]	Sweden	1992–2015	Retrospective	152	67 (44%)	0.4–19y	I, III, IV	DI, unerupted teeth, taurodontism
Bendixen (2019) [27]; Gjørup (2021) [29]; Hald (2018) [30]; Thuesen (2018) [15]	Denmark	2011–2013	Prospective	75	40 (53%)	20–77y	I, III, IV	Malocclusion, tooth agenesis, endodontically affected teeth
Clark (2019) [28]	UK	NA	Retrospective	37/92^a^	40 (43%)	8 m–18y	I, III, IV, V	DI, malocclusion, caries
De Nova-García (2022) [20]	Spain	NA	Prospective; case–control study	OI: 49; Controls: 50	OI: 25 (51%); Controls: 26 (52%)	4–18y	I, III, IV, V	Malocclusion
Elfituri (2024) [3]	Spain	2020–2022	Prospective	24/65^a^	46 (70%)	16–68y	I, III, IV	DI, malocclusion, decayed and filled teeth
Elnagdy (2012) [49]	Egypt	NA	Prospective	48	NA	NA	I, III, IV, V	DI, tooth agenesis, malocclusion, enamel hypocalcification, attrition, altered tooth position, crowding, tooth size anomalies
Kim (2013) [31]	Korea; Japan	NA	Retrospective	16^b^	7 (44%)	2 m–45y	V	DI (including pulp obliteration, pulp stones), agenesis, ectopic eruption, short roots
Lindau (1999) [32]; Lindau (1999) [33]	Norway	NA	Prospective; case–control study	OI: 15; Controls: 19	NA	Preschool children–70y	I, III, IV	DI, denticles, dentine-enamel junction abnormalities, pulpal obliteration, dental structural abnormalities
Ma (2019) [13]; Taqi (2021) [45]; Taqi (2021) [46]	Canada	2015–2017	Prospective	319	185 (58%)	2.8–75.8y	I, III, IV	DI, malocclusion, agenesis, unerupted teeth, caries, pulp obliteration, taurodontism
Maioli (2019) [34]	Italy	2008–2016	Prospective	237/340^a,b^	190 females (55.9%) 150 males (44.1%)	NA/children and adults	I, III, IV	DI
Majorana (2010) [35]	Italy	NA	Prospective	16	6 (38%)	6–12 y	I, III, IV	DI, attrition, enamel fractures
Malmgren & Lindskog (2003) [37]^¥^; Malmgren & Norngren (2002) [5]^§^	Sweden	1992–1999	Prospective; case–control study^§^	^¥^68 ^§^OI: 40; DI type II: 18; Controls: 20	NA	0.3–20y	I, III, IV	DI (including attrition and fractures), malocclusion, tooth agenesis, tooth impaction, decayed surfaces, disturbances in tooth eruption, traumatic injuries, caries; abnormalities in crown shape, cervical constrictions, pulp obliteration, taurodontism, invaginations, and denticles
Andersson (2020) [26]; Malmgren (2017) [36]	Sweden	2005–2015	Retrospective Malmgren 2017; Prospective Andersson 2017	128	54 (42%)	NA/children and adolescents	I, III, IV	Tooth agenesis (DI in patients with agenesis)
Malmgren (2021) [16]	Sweden	1991–2019	Retrospective; case–control study	OI, BP-treated: 55; OI, non-treated: 164	90 (41%)	5.7–14.7y	I, III, IV	DI, tooth agenesis, dens invaginatus and evaginatus, anomalies in tooth size and form, enamel disturbances
Marçal (2019) [38]	Brazil	2017–2018	Retrospective; case–control study	OI: 24; Controls: 48	12 (50%)	2–45y	I, IV	DI, tooth agenesis, tooth retention, tooth impaction, taurodontism, microdontia, bulbous crowns, periradicular radiolucency, residual roots, narrow and thin roots, pulp obliteration, radicular dilaceration
Martìn-Vacas (2022) [39]	Spain	2017–2018	Prospective; case–control study	OI: 17; Controls: 30	NA	NA/children	I, III, IV	DI
Mohd Nawawi (2018) [40]	Malaysia	NA	Prospective	29^b^	13 (44.8%)	NA	I, III, IV, V	DI
Nguyen (2021) [18]	Vietnam	2018–2020	Prospective	68	27 (40%)	3–17y	I, III, IV	DI, malocclusion, tooth eruption, impacted and missing teeth, caries
O'Connell & Marini (1999) [10]	USA	NA	Prospective	40^b^	25 (62.5%)	1–17.5y	III, IV	DI (including attrition and fractures), malocclusion, ectopic eruption, dental development, missing/malformed teeth, caries
Okawa (2017) [41]	Japan	NA	Retrospective	106	51 females (48.1%) 53 males (50.0%) 2 unknown (1.9%)	NA/children	I, III, IV, V, unknown	DI, malocclusion, tooth extraction
Retrouvey (2019) [42]	North America	NA	Prospective	14	10 (71%)	3–50y	V	DI, tooth agenesis, retained teeth, impacted teeth, malocclusion
Rizkallah (2013) [43]	Canada	ca. 2008–2013	Prospective; case–control study	OI: 49; Controls: 49	28 (57%)	5–19y	I, III, IV, V	Malocclusion
Sæves (2009) [14]	Norway	2003–2005	Prospective	94	54 (57%)	> 25y	I, III, IV	DI, tooth agenesis, tooth loss, malocclusion, filled teeth, endodontically treated teeth
Schwartz & Tsipouras (1984) [44]	Canada	NA	Prospective	28^b^	NA	2.5–45y	I, III, IV	DI, malocclusion, impacted teeth, attrition, pulp obliteration, caries
Vuorimies (2017) [47]	Finland	2013	Prospective; retrospective for control group; case–control study	OI, BP-treated: 22; OI, non-treated: 50; Controls: 50	9/22 (41%)	3.9–15.7y	I, III, IV	DI, tooth eruption, dental age advancement in regard to BP treatment
Yamaguti (2023) [48]	France/Brazil	NA	Retrospective in regard to clinical evaluation; prospective radiographic assessment	81	39 (48.1%)	NA	I, III, IV	DI

*BP* bisphosphonate, *DI* dentinogenesis imperfecta, *NA* not available, *n* number, *OI* osteogenesis imperfecta, *y* year

^a^If only part of the group received dental evaluation: n of individuals assessed for dental manifestations/n of total study population

^b^Some subjects were selected from the same family

### Included Studies

This review includes case series, cohort studies, and case–control studies investigating dental issues in individuals with OI. Table 1 provides an overview of the selected studies, detailing aspects, such as the countries and inclusion periods, study designs (prospective or retrospective), the number of participants screened for dental abnormalities, demographic information including age ranges and gender distributions, clinical classifications of OI in the cohorts under study, and the abnormalities examined. Studies conducted on the same cohort were combined, with the results being discussed collectively as one study (Table 1): (a) Bendixen et al. [27], Gjørup et al. [29], Hald et al. [30], Thuesen et al. [15]; (b) Lindau et al. [32], Lindau et al. [33]; (c) Ma et al. [13], Taqi et al. [45]; Taqi et al. [46]; (d) Malmgren & Lindskog [37], Malmgren & Norngren [5]; and (e) Malmgren et al. [36], Andersson et al. [26]. Subsequent sections provide a summary of the literature pertaining to various dental problems reported in OI. The prevalence of DI and that of Class I, II, and III malocclusions in OI cohorts have been summarized in Tables 2 and 3, respectively.

**Table 2:** Prevalence of dentinogenesis imperfecta in the osteogenesis imperfecta cohorts included in this review

First author (year)	Age range*, years*	Total, *n*	Females,* n* (%)	OI type, *n*				Prevalence of DI in OI, *n* (%)				
				I	III	IV	Other (V, uncertain, or unknown)	Total	I	III	IV	Other (V, uncertain, or unknown)
*Clinical, radiographic, and histological diagnosis of DI*												
Andersson (2017) [25]	0.4–19y	152	67 (44%)	96	22	34	–	73 (48%)	30 (31%)	19 (86%)	24 (71%)	–
Lindau (1999) [32]; Lindau (1999) [33]	preschool children–70y	15	NA	8	2	5	–	9 (60%)	3 (38%)	1 (50%)	5 (100%)	–
Majorana (2010) [35]	6–12y	16	6 (38%)	9	3	4	–	10 (62.5%)	4 (44%)	3 (100%)	3 (75%)	–
Martìn-Vacas (2022) [39]	mean age: 9.73y at teeth extraction	17	NA	6	7	4	–	(69.2%)	(60.0%)	(66.7%)	(100%)	
*Radiographic diagnosis of DI*												
Kim (2013) [31]	2 m–45y	16^a^	NA	–	–	–	16 OI type V	0	–	–	–	0
Marçal (2019) [38]	2–45y	24	12 (50%)	9	–	15	–	18 (75.0%)	NA	–	NA	–
*Clinical and/or radiographic diagnosis of DI*												
Clark (2019) [28]	8 m–18y	37/92^b^	NA	83	6	2	1 OI type V	9/37 (24%)	NA	NA (50%)	NA	NA
Elnagdy (2012) [49]	NA	48	NA	13	26	9	–	10 (20.4%)	1 (7.7%)	7 (26.9%)	2 (22.2%)	–
Elfituri (2024) [3]	18–68y	24	NA	9	5	10	–	8 (33.3%)	2 (22.2%)	1 (20%)	5 (50%)	–
Ma (2019) [13]; Taqi (2021) [45]; Taqi (2021) [46]	2.8–75.8y	306/319^b^	185 (58%)	150	68	101	–	124 (41%)	23 (16%)	51 (82%)	50 (54%)	–
Maioli (2019) [34]	NA/children and adults	237/340^a,b^	190 females (55.9%) 150 males (44.1%)	184	24	29	–	58 (24.5%)	31 (16.8%)	14 (58.3%)	13 (44.8%)	–
Malmgren & Lindskog (2003) [37]; Malmgren & Norngren (2002) [5]	0.3–20y	65	NA	36	15	14		27 (42%)	10 (28%)	10 (67%)	7 (50%)	–
Malmgren (2021) [16]	5.7–14.7y	219	NA	153	27	39	–	44 (20.1%)	15 (9.8%)	15 (55.6%)	14 (35.9%)	–
Mohd Nawawi (2018) [40]	NA	29^a^	13 (44.8%)	5	18	5	1 OI type V	21 (73.4%)	3 (60%)	15 (83.3%)	3 (60%)	0
Nguyen (2021) [18]	3–17y	68	27 (40%)	2	31	35	–	32 (47.1%)	0 (0%)	18 (51.4%)	14 (45.2%)	–
O'Connell & Marini (1999) [10]	1–17.5y	40^a^	25 (63%)	-	22	18	–	–	–	18/22 (82%); 8/15 (36%)	15/18 (83%); 11/17 (65%)	–
Okawa (2017) [41]	NA/children	106	51 females (48.1%) 53 males (50.0%) 2 unknown (1.9%)	32	19	14	40 unknown, 1 OI type V	70 (64%)	15 (47%)	16 (84%)	13 (93%)	23 (56.1%)
Retrouvey (2019) [42]	3–50y	14	NA	–	–	–	14 OI type V	0	–	–	–	0
Sæves (2009) [14]	> 25y	94	54 (57%)	74	8	12	–	17 (19%)	7 (9.5%)	8 (100%)	2 (16.7%)	–
Schwartz & Tsipouras (1984) [44]	2.5–45y	28^a^	NA	20	7	1	–	12 (42.9%)	8 (40.0%)	3 (42.9%)	1 (100%)	–
Bendixen (2019) [27]; Gjørup (2021) [29]; Hald (2018) [30]; Thuesen (2018) [15]	45.4 ± 14.5 y	73	NA	55	6	12	–	18 (24.7%)	1 (1.8%)	5 (83.3%)	12 (100%)	–
Vuorimies (2017) [47]	3.9–15.7y	OI, BP-treated: 22 vs OI, non-treated: 50	NA	16 vs 33	6 vs 17		–	5 (22.7%) vs 19 (38.0%)	1 (6.3%) vs 6 (18.1%)	4 (66.7%) vs 13 (76.5%)		–
Yamaguti (2023) [48]	NA	81	39 (48.1%)	19	34	15	13 OI type uncertain	44 (54.3%)	3 (15.8%)	29 (85.3%)	9 (60.0%)	3 (23.1%)

*BP* bisphosphonate, *DI* dentinogenesis imperfecta, *NA* not available, *n* number, *OI* osteogenesis imperfecta, *vs* versus, *y* year

^a^Some subjects were selected from the same family

^b^If only part of the group received dental evaluation: *n* of individuals assessed for dental manifestations/*n* of total study population

**Table 3:** Prevalence of class I, II, and III occlusions in OI cohorts assessed using Angle’s classification

Author (Year)	Class I occlusion (%)	Class II malocclusion (%)	Class III malocclusion (%)
Clark (2019) [28]	11/37 (30%)		
De Nova-García (2022)^a^ [20]	4/8 (50%)	3/8 (37.5%)	1/8 (12.5%)
De Nova-García (2022)^b^ [20]	14/41 (34.1%)	2/41 (4.9%)	25/41 (61%)
Elnagdy (2012) [49]	NA	0/48 (0%)	2/48 (4.2%)
Elfituri (2024) [3]	9/24 (38%)	6/24 (24%)	9/24 (38%)
Nguyen (2021) [18]	8/48 (17%)	0/48 (0%)	40/48 (84%)
Rizkallah (2013) [43]	12/49 (25%)	9/49 (18%)	28/49 (57%)
Schwartz & Tsipouras (1984) [44]	4/27 (14.8%)	5/27 (18.5%)	18/27 (66.7%)

*NA* not available

^a^Deciduous dentition

^b^Permanent-mixed dentition

### Dental Abnormalities in Osteogenesis Imperfecta

#### Dentinogenesis Imperfecta and Related Dental Findings

Studies investigating DI in individuals with OI revealed a wide array of diagnostic approaches and criteria used to diagnose DI. For instance, while Majorana et al. and Sæves et al. considered discoloration alone as sufficient for diagnosis, Ma et al. required a combination of color change, attrition, and altered crown shape [13, 14, 35]. Although Sæves et al. diagnosed DI solely based on clinical observations of tooth color and translucency when visible signs were present, they used radiographic indicators, including tooth morphology anomalies, to diagnose DI in individuals without clinically visible signs [14]. Schwartz & Tsipouras and Thuesen et al. emphasize the necessity of multiple manifestations for diagnosis [15, 44], while many other studies do not state explicit diagnostic criteria. Table 2 categorizes these studies based on diagnostic methods: clinical, radiographic, histological, or a combination of these.

Four studies utilized histological evaluation to diagnose DI [25, 32, 33, 39]. Majorana et al. employed histology to confirm the diagnosis of DI [35], while others used it to analyze teeth from patients with and without DI based on clinical and/or radiographic assessments. Interestingly, histological examination performed by Andersson et al. identified nineteen additional cases of DI beyond those detected by clinical and radiographic methods (44/152 (29%) versus 63/152 (41%) [25]. Martìn-Vacas et al. discovered that while clinical signs of DI were evident in 41.2% of cases, and radiographic signs in 69.2%, morphological alterations were found in all teeth via scanning electron microscopy [39]. Similarly, Lindau et al. observed irregular tubules and obliterated pulps by histological examination in all OI types, irrespective of a DI diagnosis [33]. Malmgren & Lindskog did not diagnose DI histologically but set up a dysplastic dentine score (DDS) system to evaluate dysplastic manifestations in dentin, identifying a distinct cut-off value (DDS = 23) distinguishing healthy teeth from those of OI individuals with DI [37]. Interestingly, 8/17 (47%) of OI individuals without clinical signs of DI exhibited a DDS within the range observed in OI individuals with DI. All other studies relied on clinical and radiographic assessment for DI diagnosis, except for two that solely used radiographic evaluation (Table 2) [31, 38].

The prevalence of DI exhibited significant variation, both overall and within different OI types (Table 2). Among the four studies involving over 100 participants, employing clinical and/or radiographic evaluations in the same center, the overall prevalence ranged from 20 to 48% [13, 16, 25, 34]. Specifically, the prevalence within OI type I, III, and IV ranged from 9.8 to 31%, 56 to 86%, and 36 to 71%, respectively. Notably, while Andersson et al. and Malmgren et al. focused solely on dental abnormalities in children [16, 25], Ma et al.’s study encompassed both children and adults, with participants spanning up to 75.8 years of age [13]. Maioli et al. also included both children and adults [34]. In line with the aforementioned studies, with the exception of the study by Elfituri et al. [3], OI type I demonstrated the lowest prevalence of DI across all studies, consistently showing a prevalence more than two times lower than that of individuals with OI type III or IV [34]. Among eighteen studies reporting DI prevalence for both OI type III and OI type IV individuals, DI prevalence was found to be higher in OI type III individuals compared to those with OI type IV in eleven studies (Table 2). Two studies, by Kim et al. and Retrouvey et al., focused on individuals with OI type V, conducting both clinical and radiographic assessments [31, 42]. None of these individuals showed signs of DI. Mohd Nawawi et al. also included an individual with molecularly confirmed OI type V who did not have DI [40]. In contrast, a single case of DI in an OI type V individual (without genetic confirmation) was reported in a Japanese nationwide survey [41].

In the study by Andersson et al., 122/152 (80%) of OI patients had a *COL1A1* or *COL1A2* variant known to affect the formation of the collagen type I protein. Among those with predicted structurally abnormal collagen, 46 (70%) were diagnosed with DI, whereas only 15 (27%) with a quantitative defect had DI. In patients without an identified variant in *COL1A1* or *COL1A2*, 6.9% had DI [25]. Thuesen et al. found DI in eighteen individuals, of whom 94% had a qualitative collagen defect, whereas 5.6% had a quantitative defect [15]. Maioli et al. found that 43/58 individuals with DI (74.1%) exhibited a variant in either *COL1A1* or *COL1A2*. They observed that qualitative defects were more commonly associated with the occurrence of DI compared to quantitative defects (35.6% versus 16.7%). Among patients with glycine substitutions, 21/59 (35.6%) developed DI; yet, none had DI when the substitution occurred within the first 127 amino acids of the *COL1A1* helical domain. Yamaguti et al. additionally distinguished between the two collagen type I genes. In their cohort of 81 individuals, they found that 84% of patients with a defect in *COL1A2* had DI, whereas only 36% with a defect in *COL1A1* exhibited DI [48]. These findings align with results from other studies. As the authors noted, defects in *COL1A1* were more likely to result in quantitative collagen type I defects, while defects in *COL1A2* were more likely to lead to qualitative defects. Taqi et al. did not directly address DI but noted that in their OI cohort (*n* = 154) tooth discoloration was more prevalent in individuals with certain *COL1A1* and *COL1A2* variants, particularly those with helical glycine substitutions (39% and 37%, respectively), compared to splice site variants (18%) or *COL1A1* haploinsufficiency (4%). *COL1A1* haploinsufficiency showed the lowest prevalence of pulp obliteration, a clinical sign of DI [45].

DI prevalence tended to be higher in deciduous teeth compared to permanent ones. O’Connell & Marini found that 82% and 83% of deciduous teeth in OI types III and IV patients, respectively, showed discoloration, while only 36% and 65% of permanent teeth were affected. Attrition and enamel fractures were more pronounced in yellow–brown-discolored deciduous teeth [10]. Nguyen et al. also reported higher DI rates in deciduous teeth (52%) compared to permanent dentition (44%) [18]. Andersson et al. investigated both deciduous and permanent dentitions of 40 individuals with DI; half of these showed DI symptoms in both sets of teeth, while the other half only had DI in deciduous dentition. This led them to hypothesize that individuals without DI in permanent teeth may have had DI in their deciduous dentition. No individuals showed DI signs solely in permanent teeth without any in deciduous teeth [25]. Lindau et al. observed that 71% of individuals with deciduous teeth exhibited DI, while only 50% of those with permanent teeth had the condition [33]. Maioli et al. did not specify the dentition type, but they categorized their cohort based on age. They identified DI in 18/167 adults (10.8%) and 40/174 children (23.0%) [34].

In a case–control study by Vuorimies et al., the prevalence of DI was compared between children with OI who received bisphosphonates (BP) treatment and those who did not. They found abnormal dentin in 23% (5/22) of the BP-treated group and 38% (19/50) of the untreated group. The BP-treated group had a slightly higher proportion of OI type I subjects compared to the untreated group (73% versus 66%) [47]. Similarly, Malmgren et al. conducted a case–control study assessing DI prevalence among children with OI, categorized by BP treatment and age at treatment initiation. The overall prevalence of DI was 20% (44/219), with a combined prevalence of 33% (18/55) among BP-treated groups and 16% (26/164) among the untreated group. Despite these differences, statistical analysis did not show significant disparities between the groups. Notably, OI type I predominated in the untreated group (80%), whereas in the BP-treated groups, it averaged around 40% [16].

#### Malocclusions

To assess and quantify malocclusions, orthodontists employ several scoring systems, such as the Angle’s Classification, Peer Assessment Rating (PAR) Index, and Discrepancy Index from the American Board of Orthodontics [50–52]. According to the Angle’s classification, occlusions are categorized into Class I, II, or III, depending on the position of the first permanent molars and the overall dental arrangement [53]. Class I occlusion depicts a normal relationship between the dental arches and the size of the jaws and is therefore not considered a malocclusion unless other problems are present. In contrast, Class II and Class III occlusions are always classified as malocclusions. Class II malocclusion occurs when the upper teeth and jaw significantly overlap the lower teeth and jaw. This misalignment often results in a protruding upper jaw or receding lower jaw. Class III malocclusion is characterized by the lower teeth and jaw being positioned ahead of the upper teeth and jaw [53]. Both Class II and Class III malocclusions can lead to significant functional and esthetic issues, impacting chewing, swallowing, speaking, and the overall appearance of the face [54]. Additionally, malocclusions include various types, such as open bite, cross-bite, increased overjet, and increased inverted overjet.

In the included studies, malocclusions were diagnosed using various methods, including visual examination, radiographs, and study models. Photographs aided evaluation in several studies [27, 29, 42, 43]. Sæves et al. exclusively used study models [14], also known as dental casts or impressions, while others combined models with radiographs or clinical evaluation [27, 29, 43]. In addition to panoramic radiographs [42, 43], cephalometric radiographs proved to be useful in assisting in the diagnosis of malocclusions [20, 42, 43].

The prevalence of Angle Class I, II, and III occlusions in OI cohorts varied widely, ranging from 14.8 to 50%, 0 to 37.5%, and 4.1 to 84%, respectively (Table 3) [3, 18, 20, 28, 43, 44, 49]. There is very limited information provided about the BP treatment status of these cohorts assessed for Angle Class occlusions. In the study by Clark et al., 44/92 participants (48%) received BP treatment but it remains unclear how many of these were assessed for malocclusions [28]. Similarly, Elfturi et al. reported 24/65 participants (37%) who were administered BP treatment, but no details are provided about their malocclusion status [3]. In the study by Rizkallah et al., all patients either had received or were still receiving BP treatment [43]. Overall, according to Clark et al., occlusal abnormalities were observed in 30% of the evaluated cohort, with Class III malocclusions being predominant [28]. Four out of the other six studies also reported a higher prevalence of Class III occlusions compared to Class I and II. These studies showed lower occurrences of Class I (ranging from 14.8 to 25%) and Class II (ranging from 0 to 18.5%) occlusions, while Class III malocclusions affected 57% to 84% of OI patients [3, 18, 43, 44]. However, Elnagdy et al. diagnosed Class III malocclusions in only 4.2% of individuals [49], a prevalence similar to the control group assessed by Rizkallah et al. at 4% [43]. No Class II malocclusions were documented by Nguyen et al. and Elnagdy et al. [18, 49]. The study by De Nova-García et al. demonstrated varying results in the prevalence of the three occlusion types across different dentition types. In individuals with solely deciduous dentition, Class I, II, and III occlusions were found in 50%, 37.5%, and 12.5%, respectively, whereas in those with mixed-permanent dentitions, the percentages were 34.1%, 4.9%, and 61%, respectively [20] (Table 3).

When examining the prevalence of Angle malocclusions across various types of OI, Class III consistently emerged as the most prevalent, irrespective of the OI type. Nguyen et al. and O’Connell & Marini found a remarkably high prevalence of Class III malocclusions in OI types III (89.2% and 81.8%, respectively) and IV (75% and 70.6%, respectively) [10, 18]. Nguyen et al. identified Class III malocclusion in 2/2 (100%) patients with OI type I [18]. Similarly, Schwartz, & Tsipouras observed Class III occlusal abnormalities in 10/20 (50%), 7/7 (100%), and 1/1 (100%) individuals with OI types I, III, and IV, respectively [44]. De Nova-García et al. identified Class I occlusions in the mixed-permanent dentition of 61.5% of individuals with OI type I, while Class III malocclusions were present in 77.8% and 75% of patients with OI types III and IV, respectively [20]. According to Elnagdy et al., Class III occlusal abnormalities were present in only 2/48 individuals with OI, both having OI type III (2/26; 7.7%) [49]. With the exception of the study by De Nova-García et al. and Elfituri et al. [3, 20], Class I occlusions were relatively infrequent, occurring in 0% of OI type I patients (0/2), 10.8% of OI type III patients (3/28), and 25% of OI type IV patients (5/20) according to Nguyen et al. [18]. Schwartz & Tsipouras also reported that Class I occlusions were present in 25% (5/20) of OI type I patients, but none were found in patients with OI types III and IV [44]. Class II malocclusions were notably rare, with no cases reported across 48 individuals examined by Elnagdy et al. [49] and an occurrence of 9.1% in OI type III and 15.9% in OI type IV, according to O’Connell & Marini [10]. 20% (4/20) of individuals with OI type I showed Class II malocclusion in the study by Schwartz & Tsipouras; interestingly, Class II was absent in OI types III and IV [44]. Notably, OI type V presented a distinct pattern, with Class II malocclusions being more frequent compared to other moderate-severe OI types (III and IV) [42].

Overall, cross-bite, open bite, and mandibular/increased inverted overjet were the most recurrent types of malocclusions reported in OI patients. A high prevalence of cross-bite was consistently reported among studies.

Bendixen et al. found posterior cross-bite in 88% (15/17) of study participants with moderate-severe OI (types III, IV), compared to 15% (7/52) in the mild OI (type I) subgroup [27]. Posterior cross-bite was diagnosed by De Nova-García et al. in 50% (4/8; OI types not reported) and 73% (30/41; 8/15 with OI type I or V, 14/18 with OI type III, and 8/8 with OI type IV) of OI patients with deciduous or mixed-permanent dentitions, respectively [20]. Posterior cross-bite was significantly higher in OI type III (20/28; 71.4%) than in OI type IV (5/20; 25%), according to Nguyen et al., with anterior cross-bite also being prevalent (82.1% in OI type III; 60% in OI type IV) [18]. In the study by Schwartz & Tsipouras, 16/18 patients (88.9%) with Class III malocclusions were found to exhibit posterior cross-bite, either unilateral or bilateral [44]. In contrast, this occlusal abnormality was absent in OI types I (0/13) and III (0/26) in the Egyptian cohort and present in only 1/9 patients (11.1%) with OI type IV [49]. Anterior cross-bites (50%; 7/14) and posterior cross-bites (57%; 8/14) were observed in a North American OI type V cohort [42], as well as in children with OI types III and IV, assessed by O’Connell & Marini, with a prevalence ranging from 27.3 to 47.1% [10]. Anterior cross-bite was the primary malocclusion type described by Okawa et al. (28.3%; 30/106) [41]. Additionally, anterior cross-bite was concluded to be significantly different between OI and control groups based on Discrepancy Index scores of 5.6 ± 7.0 and 0.7 ± 1.4, respectively, as reported by Rizkallah et al. [43].

Okawa et al. reported open bite in 18.9% (20/106) of the overall OI cohort [41]. Elnagdy et al. observed open bites in a small percentage of OI type I patients (7.7%; 1/13), a higher rate (30.7%; 8/26) in OI type III, and 0% (0/9) in OI type IV individuals [49]. Bendixen et al. found that anterior open bites were present in 10% (5/52) of patients with OI type I and increased to 29% (5/17) among those with OI types III and IV [27]. This aligns with O’Connell & Marini, who reported a prevalence of nearly 30% for both anterior and posterior open bites in young individuals with OI types III and IV, except for anterior open bites in OI type IV, which showed a prevalence of 17.7% [10]. Two case–control studies reported statistically significant differences in Discrepancy Index scores for the prevalence of lateral open bite between individuals with OI (7.1 to 11.1) and controls (0.3) [20, 43]. Rizkallah et al. also noted a significant difference in the scores of anterior open bite between the OI and control groups (3.7 ± 6.0 vs. 0.8 ± 3.4) [43], in contrast to De Nova-García et al., where the difference was not statistically significant in permanent-mixed dentition (3.56 ± 6.7 vs 1.66 ± 4.3) [20]. Interestingly, anterior open bite resulted as high as 37.5% in deciduous teeth [20]. Anterior and lateral open bites were documented in 43% (6/14) and 36% (5/14) of an OI type V cohort, respectively [42].

Bendixen et al. and Gjørup et al. found that mandibular overjet (increased inverted overjet) occurred more frequently in patients with OI types III/IV (50 and 64%, respectively) as opposed to those with OI type I (4% and 7%, respectively) [27, 29]. Sæves et al. reported that increased inverted overjet was present in 9.6% (8/83) of OI patients, including 4.4% with OI type I (3/68) and 33.3% (5/15) with OI types III or IV [14]. De Nova-García et al. also noted significant differences in overjet between OI patients and control individuals’ permanent-mixed dentitions, especially due to inverted overjet. The Discrepancy Index values were 5.59 on average, reaching 8.67 in OI type III, while only 2.32 in controls [20]. Wide overjet was documented by Elnagdy et al., with overall occurrence of 10.4% (2/13 with OI type I, 15.4%; 2/26 with OI type III, 7.7%; 1/9 with OI type IV, 11.1%) [49].

#### Missing Teeth

The prevalence of missing teeth was investigated in numerous studies. While many did not specify the cause—such as congenital factors (tooth agenesis), extraction, or trauma—Malmgren et al. focused specifically on tooth agenesis [16, 36]. They diagnosed it in 17% of 128 children with OI, with hypodontia in 11% and oligodontia in 6%, noting a higher prevalence in OI type III (47%) compared to types I and IV (12% and 13%, respectively) [36]. Notably, 75% of individuals with oligodontia had qualitative variants in collagen type I genes. In an additional cohort of 219 children with OI, tooth agenesis was found in 14% (with oligodontia accounting for 4%). Within subgroups, the prevalence was significantly higher in children who began BP treatment before the age of 2 years compared to both the controls and those who began treatment after the age of two [16].

Marçal et al., Taqi et al., Sæves et al., O’Connell & Marini, Nguyen et al., and Elnagdy et al. reported the prevalence of missing teeth in individuals with OI types I, III, and IV [10, 14, 18, 39, 46, 49]. One case–control study analyzed 24 individuals with OI type I/IV alongside 48 age-matched controls without OI, revealing a statistically significant higher incidence of missing teeth in both the maxilla and mandible among the OI group (maxilla: 70.8% vs. 33.3%; mandible: 66.7% vs. 41.7%). Furthermore, a statistically significant higher prevalence of DI was noted in individuals with missing teeth compared to those without [36]. Taqi et al. studied 144 OI patients and found that 61% of OI type III (88/144), 52% of OI type IV (75/144), and 11% of OI type I (16/144) individuals had one or more missing teeth, with an average of 2.4 missing teeth per patient. Interestingly, in addition to third molars, the most frequently missing teeth in the general population, OI patients were also missing first premolars, canines, first molars, and central upper incisors [46]. In a cohort of 94 adult OI patients, Sæves et al. found an average of 4.1 missing teeth per patient in OI type I and 4.2 missing teeth in OI type IV. The cause of missing teeth could not be determined accurately. Possible reasons may include extraction due to caries or malocclusion, or agenesis [14]. The OI population had fewer filled teeth than the general Norwegian population [55]. Conversely, the number of endodontically treated teeth was higher in the OI cohort compared to the general Danish population [56]. OI type I had more than twice as many endodontically treated teeth compared with OI type III and IV [14]. Bendixen et al. noted a significant difference in the mean number of natural teeth between individuals with OI type I (26.6) and OI types III and IV OI (24.2) [27]. O’Connell & Marini found that 10% of 40 children (OI type III or IV) in their study had missing teeth, including upper second premolars and lower left lateral incisors and congenital absence of second premolars and second molars [10]. In contrast, Nguyen et al. observed rare occurrences of missing teeth in their cohort of 68 children, with only one case of missing upper second premolars [18]. This was also in agreement with Elnagdy et al., who diagnosed missing teeth in only 1 individual with OI type IV within their cohort of 48 patients with OI type I, III, or IV [49].

Although DI was not observed in OI type V, Kim et al. found that 8/16 (50%) patients had single or multiple congenitally missing premolars [31]. Additionally, Retrouvey et al. reported that 6/9 (66.7%) individuals with OI type V had from one to nine missing teeth, usually premolars [42].

#### Challenges in Tooth Eruption: Retention, Impaction, Ectopic Eruption, and Unerupted Teeth

Several studies have provided insights into the prevalence of unerupted teeth in individuals with OI. Marçal et al. found a higher prevalence of tooth retention, tooth impaction, and ectopic teeth (abnormal positioning) in OI individuals compared to controls (12.5% vs. 8.3%; 41.7% vs. 16.7%; 37.5% vs. 16.7%, respectively) [38]. Taqi et al. discovered unerupted teeth in 25.6% (21/82) of OI patients, with individuals affected with OI type III exhibiting the highest prevalence (70%; 7/10), followed by OI types IV (40%; 13/32) and I (3%; 1/40). Notably, upper second molars were frequently affected. Furthermore, specific OI variants, particularly collagen α1 and α2 glycine substitutions, and early-onset BP treatment, were linked to higher incidences of unerupted teeth. Unerupted teeth were significantly more common in patients with α1 and α2 glycine variants or substitutions compared to those with quantitative defects. Early-onset BP treatment significantly increased the risk of unerupted teeth in patients with OI types III and IV (OR = 1.68, 95% CI: 1.15–1.53) [46]. Nguyen et al. noted an irregular eruption time in 14.7% of their cohort, while 85.3% had age-appropriate eruption [18]. O’Connell & Marini observed that while most patients had normal tooth eruption times for both deciduous and permanent teeth, 13/40 (32.5%) experienced ectopic eruptions of first or second molars [10]. In Schwartz & Tzipouras’s investigation, 17.8% of the 28 patients (5/28), spanning various age groups, had impacted first or second molars in their permanent dentition, with four cases associated with OI type III (4/7) and one with OI type I (1/20) [44]. Additionally, Andersson et al. found that 31% (29/93) of the children and adolescents under study exhibited retention of permanent second molars. Within this cohort, 16% (5/32) with quantitative defects and a notable 50% (21/52) with qualitative defects showed retention—a statistically significant contrast. Consequently, 69% of those diagnosed with OI type III and only 19% of those with OI type I manifested retention of their permanent second molars. Moreover, maxillary mesioangular retention was more prevalent (63%), compared to mandibular mesioangular retention (46%) [25].

In individuals with OI type V, Kim et al. observed ectopic eruption of molars in 2/16 patients (12.5%) [31], while Retrouvey et al. found that 3/9 patients had retained deciduous teeth beyond the normal range of exfoliation and 8/9 had impacted permanent teeth [42].

## Discussion

One of the key findings of this review is the confirmation of the high prevalence of dental abnormalities in OI types I, III, and IV. DI is the most reported dental abnormality; additionally, severe malocclusions, tooth agenesis, and abnormalities in tooth eruption occur frequently.

Non-syndromic DI types (types II and III) do not occur in OI and their prevalence is approximately 1 in 6000–8000 [11]. Study prevalence of DI (type I) in OI varies. Four investigations in this review, each involving over 100 individuals, have found DI in one-fifth to half of OI patients [13, 16, 34, 41]. Some larger cohort studies, which have been excluded due to insufficient data on detailed dental evaluations, suggest a possible higher prevalence of DI. For instance, Yamaguti et al. find an overall prevalence of 52.4% in 906 individuals using literature databases [48]. Zhytnik et al. diagnose DI in 54.7% of 143 Ukrainian OI individuals [57], while Wei et al. identify it in 62.1% of 116 Chinese OI individuals [58]. These cohorts’ DI diagnosis criteria are unclear (e.g., whether based solely on tooth discoloration or on patient reports without dental evaluation), complicating comparisons with the studies included in the present review. Nonetheless, these findings underscore the systemic nature of OI, as the defective collagen synthesis inherent in this condition can affect not only bone development but also the development of dental tissues. Majorana et al. propose the odontoblast dysfunction hypothesis, which suggest that abnormal dentin might result from the accumulation of abnormal procollagen [35].

Lack of standardized guidelines hinders DI diagnosis and the determination of its prevalence. No clear criteria exist for identifying sufficient indicators for DI or choosing diagnostic methods. As shown in Table 2, the prevalence of DI varies widely, likely due to these inconsistencies. The prevalence rates differ significantly depending on the diagnostic methods used [15, 39]. Several studies observe that teeth appearing normal upon visual examination might show abnormalities when assessed with radiographic or histological methods [32, 37, 39, 59]. Therefore, in certain instances, clinical evaluation alone is insufficient to exclude DI, as dentin abnormalities may also manifest through radiographic or histological features. The absence of uniform guidelines complicates the collection of accurate epidemiological data, underscoring the need for a standardized diagnostic approach to improve accuracy and comparability in DI prevalence studies. For future studies, until a standardized approach is established, it is essential to clearly document the diagnostic methods used to facilitate more accurate comparisons between studies.

In the general population, Angle Class I occlusions are the most common, with a prevalence exceeding 70% in the global population. Class II malocclusions follow, with a prevalence ranging 19.6–23%, and Class III malocclusions are the least common, with a prevalence ranging from 4 to 5.9% [60]. However, in individuals with OI, this distribution differs, with severe (Class III) malocclusions being reported most often. Schwartz & Tsipouras have suggested that severe skeletal deformities in these patients can cause severe occlusal problems [44], likely due to their impact on the jawbones. However, Class III malocclusions have also been diagnosed in OI type I patients [18, 44]. Cross-bite has an incidence in the general population of approximately 9.3–11.7% [60]. In comparison, various studies report the incidence of Class III malocclusions and cross-bite in OI cohorts ranging from 12.5 to 84% [18, 20] and 27.5 to 73.2% [10, 20], respectively, except for the study by Elnagdy et al., who have found cross-bite in only 1/48 individuals in their study [49] (Table 3). Additionally, open bite occurs more frequently in OI compared to the general population (18.8%–37.5% [20, 41], compared to 4.9–5.3% [60], respectively). In contrast, Class I occlusions appear to occur less frequently in OI compared to the general population (14.8–50.0% [20, 44] versus 73–74.7% [60], respectively). Class II malocclusions may have a similar incidence (OI: 0–37.5% [18, 20] versus general population: 19.6–23% [60]). Cephalometric assessment of malocclusions provides insight into the skeletal relationship between the mandible and maxilla. Individuals with OI often exhibit cephalometric deviations that may contribute to various malocclusions. A notable feature is relative mandibular prognathism, which is likely due to maxillary underdevelopment and/or mandibular overgrowth [20, 42, 43].

The etiology behind missing teeth—whether agenesis, extraction, or trauma—often remains unclear. Interestingly, a statistically significant association between DI and tooth agenesis has been described [36, 38]. In OI, tooth agenesis has been noted in 14–17% of individuals [16, 36], compared to 3.2–7.6% in the general population [61]. Collagen structural abnormalities may affect embryonic tooth development, especially early mineralization. This could be due to altered epithelial-mesenchymal signaling within the extracellular matrix, which may lead to arrested tooth development [36, 42, 62]. Agenesis has also been reported in OI type V, caused by *IFITM5* variants. However, the role of *IFITM5* in tooth development is unclear, especially considering its still obscure function in collagen type I regulation [31, 42].

Disturbances in tooth eruption are found in 1.6–2.3% of the general population [63]. In studies focusing on OI, various alterations have been noted. Overall, tooth eruption in OI is observed to be either age appropriate or delayed, with two studies even documenting accelerated tooth eruption [10, 47]. However, ectopic eruptions and unerupted teeth are more frequent in OI, occurring in 32.5–37.5% [10, 38] and 25.6% [46], respectively, compared to rates of 0.8–9.5% [64, 65] and 1.6–2.3% [63] reported in the general population. Collagen defects can alter the mechanical properties of dental tissues. Vourimies et al. have proposed that the increased bone turnover which characterizes OI could lead to accelerated dental development and eruption [47].

The studies in this review indicate that dental abnormalities are more common in OI types III and IV than in OI type I. Most studies show that OI type I has the lowest prevalence of DI, with rates being more than twice as low as those seen in OI type III or IV (Table 2). Consequently, investigations of OI genotype and phenotype reveal that DI is more common in individuals with qualitative defects of collagen type I compared to quantitative defects [15, 25]. Similar to DI, occlusion issues occur more commonly in OI types III and IV than OI type I [14, 18, 20, 27, 29]. Individuals with OI type V appear generally not affected by DI, although a few isolated cases have been reported [41, 58, 66]. In the studies by Retrouvey et al. and Kim et al., none of the OI type V patients exhibit DI [29, 42]. The absence of DI in these individuals might be explained by the lack of collagen type I abnormalities. In contrast, malocclusion (specifically Angle’s Class II, anterior and posterior cross-bites, as well as open bites) and missing teeth (predominantly premolars), are reported in OI type V [5, 20, 45, 58]. It should be noted that, compared to OI types I, III, and IV, the number of patients with OI type V is considerably limited. Therefore, drawing definitive conclusions is not possible, as the described dental findings may be coincidental rather than specifically associated with OI type V. Although DI is generally not reported, it is important to raise awareness of the potential presence of dental abnormalities in patients with OI type V.

Different age ranges in studies make it difficult to compare dental abnormalities by age. However, when focusing on dentition type—deciduous versus permanent teeth—DI and tooth discoloration appear more prevalent in deciduous dentition [5, 10, 18]. Additionally, yellow–brown discoloration is more frequent than gray discoloration and often affects a larger tooth surface in deciduous dentition [10, 35]. Severe attrition and enamel fractures are also more common in deciduous teeth compared to permanent teeth [5, 10, 35, 49]. Above studies are in line with Maioli et al. who differentiated between children and adults instead of dentition type. DI is found in 18/167 adults (10.8%) and 40/174 children (23.0%) [34]. Andersson et al. have found that half of 40 individuals with DI exhibited signs of DI in both deciduous and permanent teeth, while the remainder only had DI in deciduous dentition. This has led them to suggest a potential transition of DI from deciduous to permanent dentition, as none showed DI signs in permanent teeth only [25]. Lindau et al. observe that DI was more frequent among individuals with deciduous teeth than among those with permanent teeth [33]. However, this variation might be due to the higher proportion of individuals with OI type I in the permanent dentition group or to the diagnostic criteria used. De Nova-García et al. sheds light on malocclusion prevalence in individuals with OI across dentition types. Class I and Class II occlusions are less frequent in permanent-mixed teeth, while Class III malocclusions are found more often in permanent-mixed than in deciduous dentition (4.1% versus 61%). Overjet, overbite, posterior cross-bite, and anterior open bite do not show significant differences in the deciduous dentition of OI patients compared to non-OI controls, while statistically significant differences are noted in permanent-mixed dentition [20]. Regarding missing teeth, no specific data about the involved dentition type is available. In a cohort with OI type V, both deciduous and permanent dentitions are affected by retained or impacted teeth [42].

In addition to the dental abnormalities covered in this review, studies have shown a wide range of dental abnormalities that can occur in individuals with OI. This includes morphological abnormalities, enamel disturbances, short roots, caries, pulp obliteration, and attrition (Table 1). In individuals with OI, the prevalence of caries appears consistent across studies [5, 18, 28] and similar to the global prevalence (43% in deciduous teeth; 29% in permanent teeth) [67]. Interestingly, the study by Ma et al. has found a significant association between DI and caries prevalence in a large cohort of 319 individuals with OI, with higher rates in those with DI and no difference across different types of OI [13]. Several studies in the general population have reported associations between various dental abnormalities. For instance, ectopic eruption is more prevalent among young individuals with agenesis [63]. Additionally, it is associated with crowding, caries and gingivitis [41]. The uneruption of the lower molar is correlated with Class II malocclusions and tooth morphology anomalies [63], and concurrent tooth agenesis and delayed eruption of permanent teeth have been reported [68]. Given the known associations among dental abnormalities in the general population, it is not surprising that individuals with OI manifest a wide spectrum of concurrent dental anomalies.

Reports indicate that individuals with OI experience normal healing after orthognathic surgery and achieve successful outcomes with orthodontic treatments [69, 70]. Interestingly, Gleizal et al. presented a case series and literature review of a total of eleven individuals, including four with OI, who were treated with BPs and underwent orthognathic surgery. None of these patients experienced post-operative complications [69]. However, complications such as edema, ecchymosis, and hemorrhage cannot be excluded due to vessel fragility. These complications may hinder the treatment procedure and delay post-treatment recovery. Therefore, dental management in individuals with OI is feasible, provided that a thorough evaluation of the patient’s history and pre-operative assessment are carefully conducted, and necessary precautions are taken [70]. Implant placement in individuals with OI can be daunting, especially when there is very low bone mineral density (BMD) in the jaw bones, as implants need to be anchored in bone. However, a systematic review including data of 23 OI patients (with unknown BMD) showed a success rate of 94.0% with a total of 116 implants, thus suggesting dental implants may be a viable treatment option for replacing missing teeth in OI [71]. BP treatment status was known for only 5 patients, of whom 4 (17.4%) had used BPs in the past.

BP therapy is frequently used off-label in pediatric patients with OI. BPs inhibit osteoclast activity and consequently can improve bone mass [72]. However, their effects on dental health remain unclear. Only two case–control studies examine the relationship between BP therapy and DI. Both studies find no statistical difference in the prevalence of DI between the BP-treated group and the non-treated OI individuals [16, 25]. Other studies highlight potential risks associated with BP therapy [72]. Evidence suggests that early or long-term use of BP therapy might lead to an increased risk of developing tooth agenesis [16], enamel defects [16], delayed eruption [46, 73], tooth impaction [38], and pulp obliteration [38] in individuals with OI. Additionally, in vivo studies regarding the effects of BPs confirm significant dental irregularities in tooth eruption and development in rats [74, 75]. Reassuringly, early BP treatment does not contribute to tooth agenesis according to Taqi et al. 2021 [46], and no evidence of BP-related osteonecrosis of the jaws (BRONJ) is found in children with OI [41, 72, 76, 77]. Interestingly, Vuorimies et al. note that BP therapy delayed dental maturation, therefore compensating for the advanced dental development found in their OI cohort [47], while Okawa et al. suggest that BP administration does not need to be suspended in patients undergoing extraction of primary teeth [41]. Based on these contradictory findings, the potential risks—or benefits—of BP therapy on dental health should be carefully considered.

A limitation of this review is that the correlational power of the findings of some studies may be insufficient due to their small sample sizes, diverse age ranges, varying demographic profiles, and different types of OI. Due to the heterogeneity in study designs, including different diagnostic tools, no statistical testing was performed. Therefore, we encourage systematic research involving large cohort and case–control studies with comprehensive clinical and molecular characterization and uniform outcome measures. This is crucial when it comes to DI diagnosis, where standardized guidelines are lacking. Additionally, patients in these studies may have received orthodontic treatments that could have influenced the study outcomes. The lack of consistent reporting on BP treatment status in the included studies limits our ability to assess its potential impact on malocclusion development. Language limitations may have limited the scope of this review, omitting relevant research. Nonetheless, this review provides valuable insights into dental abnormalities associated with OI.

In conclusion, dental anomalies such as DI, malocclusions, missing teeth, abnormal tooth eruption and development, retention, and impaction are notably frequent in individuals with OI, posing a significant concern for their oral health. Regular dental check-ups are important for everyone, but they are especially critical for individuals with OI due to these possible dental issues [19]. Even when dental abnormalities are not immediately visible, the dentin may still be affected. Early detection is crucial, as dental issues are often interconnected, and addressing them promptly can prevent more severe complications. In the USA, the UK, and the Netherlands, approximately 65–80% of individuals receive regular dental care, indicating that regular check-ups are not a given, even in developed countries. This percentage is likely lower in countries where dental care is less accessible [78–80]. This highlights the need for increased awareness and accessibility to dental care for individuals with OI [19]. Further research is essential to deepen our understanding of the underlying mechanisms of dental abnormalities in OI. To gain a more accurate understanding of the prevalence of dental abnormalities and identify which individuals with OI are at risk, prospective cohort studies are needed. These studies should include known genotypes and detailed dental examinations. Such prospective cohort studies would be valuable for establishing evidence-based guidelines for diagnosing DI and systematically evaluating dental manifestations in this complex connective tissue disorder.

## Supplementary Information

Below is the link to the electronic supplementary material.

Supplementary file1 (DOCX 21 KB)

## Abbreviations

BMD: Bone mineral density
DI: Dentinogenesis imperfecta
OI: Osteogenesis imperfecta
PAR: Peer assessment rating
BP: Bisphosphonate

## References

[1] van Dijk FS, Sillence DO (2014) Osteogenesis imperfecta: clinical diagnosis, nomenclature and severity assessment. Am J Med Genet A 164A(6):1470–1481. 10.1002/ajmg.a.36545 24715559 10.1002/ajmg.a.36545 PMC4314691

[2] Claeys L, Storoni S, Eekhoff M, Elting M, Wisse L, Pals G, Bravenboer N, Maugeri A, Micha D (2021) Collagen transport and related pathways in osteogenesis imperfecta. Hum Genet 140(8):1121–1141. 10.1007/s00439-021-02302-2 34169326 10.1007/s00439-021-02302-2 PMC8263409

[3] Elfituri AA, De Nova MJ, Najirad M (2024) The impact of osteogenesis imperfecta severity on oral health-related quality of life in Spain: a cross-sectional study. Orphanet J Rare Dis 19(1):108. 10.1186/s13023-024-03096-y 38459573 10.1186/s13023-024-03096-y PMC10921673

[4] Patel RM, Nagamani SC, Cuthbertson D, Campeau PM, Krischer JP, Shapiro JR, Steiner RD, Smith PA, Bober MB, Byers PH, Pepin M, Durigova M, Glorieux FH, Rauch F, Lee BH, Hart T, Sutton VR (2015) A cross-sectional multicenter study of osteogenesis imperfecta in North America—results from the linked clinical research centers. Clin Genet 87(2):133–140. 10.1111/cge.12409 24754836 10.1111/cge.12409 PMC5529599

[5] Malmgren B, Norgren S (2002) Dental aberrations in children and adolescents with osteogenesis imperfecta. Acta Odontol Scand 60(2):65–71. 10.1080/000163502753509446 12020117 10.1080/000163502753509446

[6] D’souza Z, Chettiankandy TJ, Ahire MS, Thakur A, Sonawane SG, Sinha A (2019) Collagen—structure, function and distribution in orodental tissues. J Global Oral Health 2(2):134–139. 10.25259/JGOH_4_2020

[7] Goldberg M, Kulkarni AB, Young M, Boskey A (2011) Dentin: structure, composition and mineralization. Front Biosci (Elite Ed) 3(2):711–735. 10.2741/e281 21196346 10.2741/e281 PMC3360947

[8] Lukinmaa PL, Waltimo J (1992) Immunohistochemical localization of types I, V, and VI collagen in human permanent teeth and periodontal ligament. J Dent Res 71(2):391–397. 10.1177/00220345920710020801 1556297 10.1177/00220345920710020801

[9] Prado HV, Teixeira SA, Rabello F, Vargas-Ferreira F, Borges-Oliveira AC, Abreu LG (2022) Malocclusion in individuals with osteogenesis imperfecta: a systematic review and meta-analysis. Oral Dis 28(2):314–325. 10.1111/odi.13715 33222339 10.1111/odi.13715

[10] O’Connell AC, Marini JC (1999) Evaluation of oral problems in an osteogenesis imperfecta population. Oral Surg Oral Med Oral Pathol Oral Radiol Endod 87(2):189–196. 10.1016/s1079-2104(99)70272-6 10052375 10.1016/s1079-2104(99)70272-6

[11] Barron MJ, McDonnell ST, Mackie I, Dixon MJ (2008) Hereditary dentine disorders: dentinogenesis imperfecta and dentine dysplasia. Orphanet J Rare Dis 20(3):31. 10.1186/1750-1172-3-31 10.1186/1750-1172-3-31 PMC2600777 19021896

[12] Shields ED, Bixler D, el-Kafrawy AM (1973) A proposed classification for heritable human dentine defects with a description of a new entity. Arch Oral Biol 18(4):543–553. 10.1016/0003-9969(73)90075-7 4516067 10.1016/0003-9969(73)90075-7

[13] Ma MS, Najirad M, Taqi D, Retrouvey JM, Tamimi F, Dagdeviren D, Glorieux FH, Lee B, Sutton VR, Rauch F, Esfandiari S (2019) Caries prevalence and experience in individuals with osteogenesis imperfecta: a cross-sectional multicenter study. Spec Care Dentist 39(2):214–219. 10.1111/scd.12368 30758072 10.1111/scd.12368 PMC6402806

[14] Saeves R, Lande Wekre L, Ambjørnsen E, Axelsson S, Nordgarden H, Storhaug K (2009) Oral findings in adults with osteogenesis imperfecta. Spec Care Dent 29(2):102–108. 10.1111/j.1754-4505.2008.00070.x 10.1111/j.1754-4505.2008.00070.x 19284510

[15] Thuesen KJ, Gjørup H, Hald JD, Schmidt M, Harsløf T, Langdahl B, Haubek D (2018) The dental perspective on osteogenesis imperfecta in a Danish adult population. BMC Oral Health 18(1):175. 10.1186/s12903-018-0639-7 30355314 10.1186/s12903-018-0639-7 PMC6201594

[16] Malmgren B, Thesleff I, Dahllöf G, Åström E, Tsilingaridis G (2021) Abnormalities in tooth formation after early bisphosphonate treatment in children with osteogenesis imperfecta. Calcif Tissue Int 109(2):121–131. 10.1007/s00223-021-00835-2 33743023 10.1007/s00223-021-00835-2 PMC8273054

[17] Kim JW, Simmer JP (2007) Hereditary dentin defects. J Dent Res 86(5):392–399. 10.1177/154405910708600502 17452557 10.1177/154405910708600502

[18] Nguyen HTT, Vu DC, Nguyen DM, Dang QD, Tran VK, Le H, Tong SM (2021) Dentinogenesis imperfecta and caries in osteogenesis imperfecta among vietnamese children. Dent J (Basel) 9(5):49. 10.3390/dj9050049 33925433 10.3390/dj9050049 PMC8144955

[19] Blokland L, Arponen H, Ahmad A, Colijn S, Gjørup H, John R, Li M, Mekking D, Parekh S, Retrouvey JM, Stutz Steiger T, Zhou L, Andersson K (2024) A standard set of outcome measures for the comprehensive assessment of oral health and occlusion in individuals with osteogenesis imperfecta. Orphanet J Rare Dis 19:294. 10.1186/s13023-024-03308-5 39138478 10.1186/s13023-024-03308-5 PMC11320983

[20] De Nova-García MJ, Bernal-Barroso F, Mourelle-Martínez MR, Gallardo-López NE, Diéguez-Pérez M, Feijoo-García G, Burgueño-Torres L (2022) Evaluation of the severity of malocclusion in children with osteogenesis imperfecta. J Clin Med 11(16):4862. 10.3390/jcm11164862 36013101 10.3390/jcm11164862 PMC9410483

[21] Glorieux FH, Rauch F, Plotkin H, Ward L, Travers R, Roughley P, Lalic L, Glorieux DF, Fassier F, Bishop NJ (2000) Type V osteogenesis imperfecta: a new form of brittle bone disease. J Bone Miner Res 15(9):1650–1658. 10.1359/jbmr.2000 10976985 10.1359/jbmr.2000.15.9.1650

[22] Unger S, Ferreira CR, Mortier GR, Ali H, Bertola DR, Calder A, Cohn DH, Cormier-Daire V, Girisha KM, Hall C, Krakow D, Makitie O, Mundlos S, Nishimura G, Robertson SP, Savarirayan R, Sillence D, Simon M, Sutton VR, Warman ML, Superti-Furga A (2023) Nosology of genetic skeletal disorders: 2023 revision. Am J Med Genet A 191(5):1164–1209. 10.1002/ajmg.a.63132 36779427 10.1002/ajmg.a.63132 PMC10081954

[23] Page M, McKenzie JE, Bossuyt PM, Boutron I, Hofmann TC, Mulrow CD et al (2021) The PRISMA 2020 statement: an updated guideline for reporting systematic reviews. PLOS Med 18(3):e1003583. 10.1371/journal.pmed.1003583 33780438 10.1371/journal.pmed.1003583 PMC8007028

[24] Study Quality Assessment Tools | NHLBI, NIH. https://www.nhlbi.nih.gov/health-topics/study-quality-assessment-tools. Accessed 2 May 2024

[25] Andersson K, Dahllöf G, Lindahl K, Kindmark A, Grigelioniene G, Åström E, Malmgren B (2017) Mutations in COL1A1 and COL1A2 and dental aberrations in children and adolescents with osteogenesis imperfecta—a retrospective cohort study. PLoS ONE 12(5):e0176466. 10.1371/journal.pone.0176466 28498836 10.1371/journal.pone.0176466 PMC5428910

[26] Andersson K, Malmgren B, Åström E, Nordgren A, Taylan F, Dahllöf G (2020) Mutations in COL1A1/A2 and CREB3L1 are associated with oligodontia in osteogenesis imperfecta. Orphanet J Rare Dis 15(1):80. 10.1186/s13023-020-01361-4 32234057 10.1186/s13023-020-01361-4 PMC7110904

[27] Bendixen KH, Gjørup H, Baad-Hansen L, Dahl Hald J, Harsløf T, Schmidt MH, Langdahl BL, Haubek D (2018) Temporomandibular disorders and psychosocial status in osteogenesis imperfecta - a cross-sectional study. BMC Oral Health 18(1):35. 10.1186/s12903-018-0497-3 29514671 10.1186/s12903-018-0497-3 PMC5842569

[28] Clark R, Burren CP, John R (2019) Challenges of delivery of dental care and dental pathologies in children and young people with osteogenesis imperfecta. Eur Arch Paediatr Dent 20(5):473–480. 10.1007/s40368-019-00424-w 30868445 10.1007/s40368-019-00424-w

[29] Gjørup H, Beck-Nielsen SS, Hald JD, Haubek D (2021) Oral health-related quality of life in X-linked hypophosphataemia and osteogenesis imperfecta. J Oral Rehabil 48(2):160–168. 10.1111/joor.13114 33058298 10.1111/joor.13114 PMC7839549

[30] Hald JD, Folkestad L, Swan CZ, Wanscher J, Schmidt M, Gjørup H, Haubek D, Leonhard CH, Larsen DA, Hjortdal JØ, Harsløf T, Duno M, Lund AM, Jensen JB, Brixen K, Langdahl B (2018) Osteogenesis imperfecta and the teeth, eyes, and ears-a study of non-skeletal phenotypes in adults. Osteoporos Int 29(12):2781–2789. 10.1007/s00198-018-4663-x 30143849 10.1007/s00198-018-4663-x

[31] Kim OH, Jin DK, Kosaki K, Kim JW, Cho SY, Yoo WJ, Choi IH, Nishimura G, Ikegawa S, Cho TJ (2013) Osteogenesis imperfecta type V: clinical and radiographic manifestations in mutation confirmed patients. Am J Med Genet A 161A(8):1972–1979. 10.1002/ajmg.a.36024 23804581 10.1002/ajmg.a.36024

[32] Lindau BM, Dietz W, Hoyer I, Lundgren T, Storhaug K, Norén JG (1999) Morphology of dental enamel and dentine-enamel junction in osteogenesis imperfecta. Int J Paediatr Dent 9(1):13–21. 10.1046/j.1365-263x.1999.00101.x 10336712 10.1046/j.1365-263x.1999.00101.x

[33] Lindau B, Dietz W, Lundgren T, Storhaug K, Norén JG (1999) Discrimination of morphological findings in dentine from osteogenesis imperfecta patients using combinations of polarized light microscopy, microradiography and scanning electron microscopy. Int J Paediatr Dent 9(4):253–261. 10.1111/j.1365-263x.1999.00143.x 10815583 10.1111/j.1365-263x.1999.00143.x

[34] Maioli M, Gnoli M, Boarini M, Tremosini M, Zambrano A, Pedrini E, Mordenti M, Corsini S, D’Eufemia P, Versacci P, Celli M, Sangiorgi L (2019) Genotype-phenotype correlation study in 364 osteogenesis imperfecta Italian patients. Eur J Hum Genet 27(7):1090–1100. 10.1038/s41431-019-0373-x 30886339 10.1038/s41431-019-0373-x PMC6777444

[35] Majorana A, Bardellini E, Brunelli PC, Lacaita M, Cazzolla AP, Favia G (2010) Dentinogenesis imperfecta in children with osteogenesis imperfecta: a clinical and ultrastructural study. Int J Paediatr Dent 20(2):112–118. 10.1111/j.1365-263X.2010.01033.x 20384825 10.1111/j.1365-263X.2010.01033.x

[36] Malmgren B, Andersson K, Lindahl K, Kindmark A, Grigelioniene G, Zachariadis V, Dahllöf G, Åström E (2017) Tooth agenesis in osteogenesis imperfecta related to mutations in the collagen type I genes. Oral Dis 23(1):42–49. 10.1111/odi.12568 27510842 10.1111/odi.12568

[37] Malmgren B, Lindskog S (2003) Assessment of dysplastic dentin in osteogenesis imperfecta and dentinogenesis imperfecta. Acta Odontol Scand 61(2):72–80. 10.1080/00016350310001398 12790503 10.1080/00016350310001398

[38] Marçal FF, Ribeiro EM, Costa FWG, Fonteles CSR, Teles GS, de Barros Silva PG, Chaves Junior CM, Ribeiro TR (2019) Dental alterations on panoramic radiographs of patients with osteogenesis imperfecta in relation to clinical diagnosis, severity, and bisphosphonate regimen aspects: a STROBE-compliant case-control study. Oral Surg Oral Med Oral Pathol Oral Radiol 128(6):621–630. 10.1016/j.oooo.2019.07.001 31399368 10.1016/j.oooo.2019.07.001

[39] Martín-Vacas A, de Nova MJ, Sagastizabal B, García-Barbero ÁE, Vera-González V (2022) Morphological study of dental structure in dentinogenesis imperfecta type I with scanning electron microscopy. Healthcare (Basel) 10(8):1453. 10.3390/healthcare10081453 36011110 10.3390/healthcare10081453 PMC9408206

[40] Mohd Nawawi N, Selveindran NM, Rasat R, Chow YP, Abdul Latiff Z, Syed Zakaria SZ, Jamal R, Abdul Murad NA, Abd Aziz BB (2018) Genotype-phenotype correlation among Malaysian patients with osteogenesis imperfecta. Clin Chim Acta 484:141–147. 10.1016/j.cca.2018.05.048 29807018 10.1016/j.cca.2018.05.048

[41] Okawa R, Kubota T, Kitaoka T, Kokomoto K, Ozono K, Nakano K (2017) Oral manifestations of Japanese patients with osteogenesis imperfecta. Pediatr Dent J 27:73–78. 10.1016/j.pdj.2017.02.001

[42] Retrouvey JM, Taqi D, Tamimi F, Dagdeviren D, Glorieux FH, Lee B, Hazboun R, Krakow D, Sutton VR (2019) Members of the BBD consortium. Oro-dental and cranio-facial characteristics of osteogenesis imperfecta type V. Eur J Med Genet 62(12):103606. 10.1016/j.ejmg.2018.12.011 30593885 10.1016/j.ejmg.2018.12.011 PMC6594916

[43] Rizkallah J, Schwartz S, Rauch F, Glorieux F, Vu DD, Muller K, Retrouvey JM (2013) Evaluation of the severity of malocclusions in children affected by osteogenesis imperfecta with the peer assessment rating and discrepancy indexes. Am J Orthod Dentofacial Orthop 143(3):336–341. 10.1016/j.ajodo.2012.10.016 23452967 10.1016/j.ajodo.2012.10.016

[44] Schwartz S, Tsipouras P (1984) Oral findings in osteogenesis imperfecta. Oral Surg Oral Med Oral Pathol 57(2):161–167. 10.1016/0030-4220(84)90206-8 6583624 10.1016/0030-4220(84)90206-8

[45] Taqi D, Moussa H, Schwinghamer T, Ducret M, Dagdeviren D, Retrouvey JM, Rauch F, Tamimi F (2021) Members of the BBDC. Osteogenesis imperfecta tooth level phenotype analysis: cross-sectional study. Bone 147:115917. 10.1016/j.bone.2021.115917 33741542 10.1016/j.bone.2021.115917 PMC8278321

[46] Taqi D, Moussa H, Schwinghamer T, Vieira AR, Dagdeviren D, Retrouvey JM, Rauch F, Tamimi F (2021) Members of the BBDC. Missing and unerupted teeth in osteogenesis imperfecta. Bone 150:116011. 10.1016/j.bone.2021.116011 34020077 10.1016/j.bone.2021.116011 PMC12211518

[47] Vuorimies I, Arponen H, Valta H, Tiesalo O, Ekholm M, Ranta H, Evälahti M, Mäkitie O, Waltimo-Sirén J (2017) Timing of dental development in osteogenesis imperfecta patients with and without bisphosphonate treatment. Bone 94:29–33. 10.1016/j.bone.2016.10.004 27725317 10.1016/j.bone.2016.10.004

[48] Yamaguti PM, de La Dure-Molla M, Monnot S, Cardozo-Amaya YJ, Baujat G, Michot C, Fournier BPJ, Riou MC, Caldas Rosa ECC, Soares de Lima Y, Dos Santos PAC, Alcaraz G, Guerra ENS, Castro LC, de Oliveira SF, Pogue R, Berdal A, de Paula LM, Mazzeu JF, Cormier-Daire V, Acevedo AC (2023) Unequal impact of COL1A1 and COL1A2 variants on dentinogenesis imperfecta. J Dent Res 102(6):616–625. 10.1177/00220345231154569 36951356 10.1177/00220345231154569

[49] Elnagdy GMHA, ElRefaiey MI, Aglan M, Ibrahim RO, Badry THM (2012) Oro-dental manifestations in different types of osteogenesis imperfecta. Aust J Basic Appl Sci 6(12):464–473

[50] Angle EH, Louis ST (1899) Classification of malocclusion. Dent Cosm 41:248–264

[51] Richmond S, Shaw WC, O’Brien KD, Buchanan IB, Jones R, Stephens CD, Roberts CT, Andrews M (1992) The development of the PAR Index (peer assessment rating): reliability and validity. Eur J Orthod 14(2):125–139. 10.1093/ejo/14.2.125 1582457 10.1093/ejo/14.2.125

[52] Casko JS, Vaden JL, Kokich VG, Damone J, James RD, Cangialosi TJ, Riolo ML, Owens SE Jr, Bills ED (1998) Objective grading system for dental casts and panoramic radiographs. American board of orthodontics. Am J Orthod Dentofacial Orthop 114(5):589–599. 10.1016/s0889-5406(98)70179-9 9810056 10.1016/s0889-5406(98)70179-9

[53] Kanas RJ, Carapezza L, Kanas SJ (2008) Treatment classification of class III malocclusion. J Clin Pediatr Dent 33(2):175–185. 10.17796/jcpd.33.2.431877341u182416 19358388 10.17796/jcpd.33.2.431877341u182416

[54] Lombardo G, Vena F, Negri P, Pagano S, Barilotti C, Paglia L, Colombo S, Orso M, Cianetti S (2020) Worldwide prevalence of malocclusion in the different stages of dentition: a systematic review and meta-analysis. Eur J Paediatr Dent 21(2):115–122. 10.23804/ejpd.2020.21.02.05 32567942 10.23804/ejpd.2020.21.02.05

[55] Schuller AA, Holst D (1998) Changes in the oral health of adults from Trøndelag, Norway, 1973–1983-1994. Community Dent Oral Epidemiol 26(3):201–208. 10.1111/j.1600-0528.1998.tb01950.x 9669599 10.1111/j.1600-0528.1998.tb01950.x

[56] Kirkevang LL, Hörsted-Bindslev P, Ørstavik D, Wenzel A (2001) Frequency and distribution of endodontically treated teeth and apical periodontitis in an urban Danish population. Int Endodontic J 34(3):198–205. 10.1046/j.1365-2591.2001.00370.x 10.1046/j.1365-2591.2001.00370.x 12193265

[57] Zhytnik L, Maasalu K, Pashenko A, Khmyzov S, Reimann E, Prans E, Kõks S, Märtson A (2019) COL1A1/2 pathogenic variants and phenotype characteristics in Ukrainian osteogenesis imperfecta patients. Front Genet 9(10):722. 10.3389/fgene.2019.00722 31447884 10.3389/fgene.2019.00722 PMC6696896

[58] Wei S, Yao Y, Shu M, Gao L, Zhao J, Li T, Wang Y, Xu C (2022) Genotype-phenotype relationship and follow-up analysis of a Chinese cohort with osteogenesis imperfecta. Endocr Pract 28(8):760–766. 10.1016/j.eprac.2022.05.003 35550181 10.1016/j.eprac.2022.05.003

[59] Lygidakis NA, Smith R, Oulis CJ (1996) Scanning electron microscopy of teeth in osteogenesis imperfecta type I. Oral Surg Oral Med Oral Pathol Oral Radiol Endod 81(5):567–572. 10.1016/s1079-2104(96)80048-5 8734703 10.1016/s1079-2104(96)80048-5

[60] Alhammadi MS, Halboub E, Fayed MS, Labib A, El-Saaidi C (2018) Global distribution of malocclusion traits: a systematic review. Dent Press J Orthod 23(6):40.e1-40.e10. 10.1590/2177-6709.23.6.40.e1-10.onl 10.1590/2177-6709.23.6.40.e1-10.onl PMC6340198 30672991

[61] Polder BJ, Van’t Hof MA, Van der Linden FP, Kuijpers-Jagtman AM (2004) A meta-analysis of the prevalence of dental agenesis of permanent teeth. Community Dent Oral Epidemiol 32(3):217–226. 10.1111/j.1600-0528.2004.00158.x 15151692 10.1111/j.1600-0528.2004.00158.x

[62] Juuri E, Balic A (2017) The biology underlying abnormalities of tooth number in humans. J Dent Res 96(11):1248–1256. 10.1177/0022034517720158 28783411 10.1177/0022034517720158

[63] Bondemark L, Tsiopa J (2007) Prevalence of ectopic eruption, impaction, retention and agenesis of the permanent second molar. Angle Orthod 77(5):773–778. 10.2319/072506-306.1 17685771 10.2319/072506-306.1

[64] Güven Y (2018) Prevalence of ectopic eruption of first permanent molars in a Turkish population. Eur Oral Res 52(1):1–5. 10.26650/eor.2018.45227 30574592 10.26650/eor.2018.45227 PMC6300125

[65] Haghighi S, Gharekhani S, Abesi F, Ghasempour M, Hajian-Tilaki K (2023) Prevalence of ectopic maxillary canine and its association with other dental anomalies in children: an observational study. Eastern J Med 28:133–138. 10.5505/ejm.2023.27037

[66] Cao YJ, Wei Z, Zhang H, Zhang ZL (2019) Expanding the clinical spectrum of osteogenesis imperfecta type V: 13 additional patients and review. Front Endocrinol (Lausanne) 12(10):375. 10.3389/fendo.2019.00375 10.3389/fendo.2019.00375 PMC6581704 31244780

[67] World Health Organization (2022) Global oral health status report: Towards universal health coverage for oral health by 2030. Executive Summary. World Health Organization, Geneva

[68] Gelbrich B, Hirsch A, Dannhauer KH, Gelbrich G (2015) Agenesis of second premolars and delayed dental maturation. J Orofac Orthop 76(4):338–350. 10.1007/s00056-015-0295-3 26141045 10.1007/s00056-015-0295-3

[69] Gleizal A, Meon A, Asselborn M, Chauvel-Picard J (2023) Orthognathic surgery in patients treated with bisphosphonates: a case series. J Craniomaxillofac Surg 51(9):521–527. 10.1016/j.jcms.2023.06.002 37460349 10.1016/j.jcms.2023.06.002

[70] Kim DY, Baik U, Jeon JH (2020) Osteogenesis imperfecta and combined orthodontics and orthognathic surgery: a case report on two siblings. J Korean Assoc Oral Maxillofac Surg 46(1):70–77. 10.5125/jkaoms.2020.46.1.70 32158684 10.5125/jkaoms.2020.46.1.70 PMC7049766

[71] Oelerich O, Kleinheinz J, Bohner L, Wiesmüller V, Hanisch M (2022) Dental implants in people with osteogenesis imperfecta: a systematic review. Int J Environ Res Public Health 19(3):1563. 10.3390/ijerph19031563 35162583 10.3390/ijerph19031563 PMC8835393

[72] Bhatt RN, Hibbert SA, Munns CF (2014) The use of bisphosphonates in children: review of the literature and guidelines for dental management. Aust Dent J 59(1):9–19. 10.1111/adj.12140 24495226 10.1111/adj.12140

[73] Kamoun-Goldrat A, Ginisty D, Le Merrer M (2008) Effects of bisphosphonates on tooth eruption in children with osteogenesis imperfecta. Eur J Oral Sci 116(3):195–198. 10.1111/j.1600-0722.2008.00529.x 18471236 10.1111/j.1600-0722.2008.00529.x

[74] Bradaschia-Correa V, Massa LF, Arana-Chavez VE (2007) Effects of alendronate on tooth eruption and molar root formation in young growing rats. Cell Tissue Res 330(3):475–485. 10.1007/s00441-007-0499-y 17901984 10.1007/s00441-007-0499-y

[75] Hiraga T, Ninomiya T, Hosoya A, Nakamura H (2010) Administration of the bisphosphonate zoledronic acid during tooth development inhibits tooth eruption and formation and induces dental abnormalities in rats. Calcif Tissue Int 86(6):502–510. 10.1007/s00223-010-9366-z 20411381 10.1007/s00223-010-9366-z

[76] Contaldo M, Luzzi V, Ierardo G, Raimondo E, Boccellino M, Ferati K, Bexheti-Ferati A, Inchingolo F, Di Domenico M, Serpico R, Polimeni A, Bossù M (2020) Bisphosphonate-related osteonecrosis of the jaws and dental surgery procedures in children and young people with osteogenesis imperfecta: a systematic review. J Stomatol Oral Maxillofac Surg 121(5):556–562. 10.1016/j.jormas.2020.03.003 32156673 10.1016/j.jormas.2020.03.003

[77] Malmgren B, Aström E, Söderhäll S (2008) No osteonecrosis in jaws of young patients with osteogenesis imperfecta treated with bisphosphonates. J Oral Pathol Med 37(4):196–200. 10.1111/j.1600-0714.2007.00607.x 18321345 10.1111/j.1600-0714.2007.00607.x

[78] GOV.UK. Adult Oral Health Survey 2021: Service Use and Barriers to Accessing Care. 2021. https://www.gov.uk/government/statistics/adult-oral-health-survey-2021/adult-oral-health-survey-2021-service-use-and-barriers-to-accessing-care. Accessed 17 Jul 2024

[79] Cha A, Cohen R (2022) Dental care utilization among adults aged 18−64: United States 2019 and 2020. National Center for Health Statistics (U.S.). 10.15620/cdc:115597 35575758

[80] Centraal Bureau voor Statistiek, «Gezondheid, leefstijl, zorggebruik en -aanbod, doodsoorzaken; vanaf 1900. https://opendata.cbs.nl/#/CBS/nl/dataset/37852/table?ts=1721232828617. Accessed 17 Jul 2024

