# Supplementary material for: Dental Abnormalities in Osteogenesis Imperfecta: A Systematic Review
Source: Calcif Tissue Int. 2024 Sep 18;115(5):461–79. doi: 10.1007/s00223-024-01293-2 (PMC11531448; doi:10.1007/s00223-024-01293-2)
Supplement: Supplementary file 1 — Supplementary file1 (DOCX 21 KB) [file 223_2024_1293_MOESM1_ESM.docx]

# *Calcified Tissue International*

# **Dental Abnormalities in Osteogenesis Imperfecta: a Systematic Review**

Laura Ventura^1,2,3,4§^[
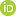
](https://orcid.org/0009-0005-1732-6618), Sara J.E. Verdonk^3,4,5§^[
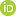
](https://orcid.org/0000-0001-5992-6789), Lidiia Zhytnik^1,2,3,4^[
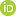
](https://orcid.org/0000-0003-4682-0402), Angela Ridwan-Pramana^6,7^, Marjolijn Gilijamse^3,4,6^[
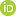
](https://orcid.org/0000-0003-0609-9227), Willem H. Schreuder^4,6,8^, Kirsten A. van Gelderen-Ziesemer^9^, Ton Schoenmaker^3,4,10¥^[
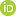
](https://orcid.org/0000-0001-6649-374X), Dimitra Micha^1,2,3,4¥^[
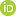
](https://orcid.org/0000-0001-7890-4411), Elisabeth M.W. Eekhoff^2,3,4,5¥*^[
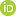
](https://orcid.org/0000-0001-5399-676X)

*^1^ Department of Human Genetics, Amsterdam UMC Location Vrije Universiteit Amsterdam, Amsterdam, The Netherlands*

*^2^ Amsterdam Reproduction and Development, Amsterdam, The Netherlands*

*^3^ Amsterdam Movement Sciences, Amsterdam, The Netherlands*

*^4^ Amsterdam Bone Center, Amsterdam, The Netherlands*

*^5^ Department of Internal Medicine Section Endocrinology, Amsterdam UMC Location Vrije Universiteit Amsterdam, De Boelelaan 1117, Amsterdam, The Netherlands*

*^6^ Department of Oral and Maxillofacial Surgery, Amsterdam UMC Location Vrije Universiteit Amsterdam, Amsterdam, The Netherlands*

*^7^ Department Maxillofacial Prosthodontics, Stichting Bijzondere Tandheelkunde, Amsterdam, The Netherlands*

*^8^ Department of Oral Diseases and Maxillofacial Surgery, Academic Centre for Dentistry Amsterdam (ACTA), Amsterdam, The Netherlands*

*^9^ Medical Library, Vrije Universiteit, Amsterdam, The Netherlands*

*^10^ Department of Periodontology, Academic Centre for Dentistry Amsterdam (ACTA), Amsterdam, The Netherlands*

*^§^ These authors contributed equally and share first authorship*

*^¥^ These authors contributed equally and share last authorship*

** Corresponding author: emw.eekhoff@amsterdamumc.nl*

# **Table S1. Search strategy. Pubmed, Embase, and Web of Science databases were searched for articles up to June 6, 2024.**

**Ovid/Medline Results (June 6, 2024)**

| **Search** | **Ovid/Medline Query – June 6, 2024** | **Results** |
| --- | --- | --- |
| #3 | exp Osteogenesis Imperfecta/ or (brittle-bone* or bruck* or fibrogenesis-imperfecta or osteopsathyros* or lobstein* or vrolik* or periostal-aplasia or osteogenesis-imperfect* or fragilitas-oss*).ti,ab,kf. | 7466 |
| #2 | exp Dentinogenesis Imperfecta/ or ((dental or dentition or dentin* or tooth or teeth or enamel or molar or odontogenesis) adj3 (abonormal* or anomal* or malform* or deformit* or dysplasia* or imperfect*)).ti,ab,kf. | 5343 |
| #1 | 1 and 2 | 368 |

**Embase.com Results (June 6, 2024)**

| **Search** | **Embase Query – June 6, 2024** | **Results** |
| --- | --- | --- |
| #4 | #3 NOT 'conference abstract'/it | 497 |
| #3 | #1 AND #2 | 590 |
| #2 | 'tooth malformation'/exp OR (((dental OR dentition OR dentin* OR tooth OR teeth OR enamel OR molar OR odontogenesis) NEAR/3 (abonormal* OR anomal* OR malform* OR deformit* OR dysplasia* OR imperfect*)):ti,ab,kw) | 20627 |
| #1 | 'osteogenesis imperfecta'/exp OR 'brittle bone*':ti,ab,kw OR bruck*:ti,ab,kw OR 'fibrogenesis imperfecta':ti,ab,kw OR osteopsathyros*:ti,ab,kw OR lobstein*:ti,ab,kw OR vrolik*:ti,ab,kw OR 'periostal aplasia':ti,ab,kw OR 'osteogenesis imperfect*':ti,ab,kw OR 'fragilitas oss*':ti,ab,kw | 11289 |

**Clarivate Analytics/Web of Science Core Collection Results (June 6, 2024)**

| **Search** | **Web of Science Query – June 6, 2024** | **Results** |
| --- | --- | --- |
| #1 | TS=(brittle-bone* OR bruck* OR fibrogenesis-imperfecta OR osteopsathyros* OR lobstein* OR vrolik* OR periostal-aplasia OR osteogenesis-imperfect* OR fragilitas-oss*) | 11066 |
| #2 | TS=((dental OR dentition OR dentin* OR tooth OR teeth OR enamel OR molar OR odontogenesis) NEAR/3 (abonormal* OR anomal* OR malform* OR deformit* OR dysplasia* OR imperfect*)) | 5220 |
| #3 | #2 AND #1 | 330 |

**Results:**

Ovid/Medline

Inception – **(June 6, 2024)**

368

Embase.com

Inception – **(June 6, 2024)**

497

Clarivate Analytics/Web of Science Core Collection

Inception – **(June 6, 2024)**

330

Number of Non-Duplicate Citations: 1195
